# Iron bioavailability of maize (*Zea mays* L.) after removing the germ fraction

**DOI:** 10.3389/fpls.2023.1114760

**Published:** 2023-03-07

**Authors:** Johanna I. Keigler, Jason A. Wiesinger, Sherry A. Flint-Garcia, Raymond P. Glahn

**Affiliations:** ^1^ Untied States Department of Agriculture - Agriculture Research Services (USDA-ARS), Robert W. Holley Center for Agriculture and Health, Ithaca, NY, United States; ^2^ Untied States Department of Agriculture - Agriculture Research Services, Plant Genetics Research Unit, Columbia, MO, United States

**Keywords:** genetic characterization, maize (Zea mays L.), degermination, iron bioavailability, phytate, zinc, polyphenols

## Abstract

Maize is a staple food for many communities with high levels of iron deficiency anemia. Enhancing the iron concentrations and iron bioavailability of maize with traditional breeding practices, especially after cooking and processing, could help alleviate iron deficiency in many of these regions. Previous studies on a small number of maize genotypes and maize flour products indicated that degermination (germ fraction removed with processing) could improve the iron bioavailability of maize. This study expanded upon this research by evaluating the iron bioavailability, mineral concentrations, and phytate concentrations of 52 diverse maize genotypes before (whole kernels) and after degermination. Whole and degerminated maize samples were cooked, dried, and milled to produce corn flour. Iron bioavailability was evaluated with an *in vitro* digestion Caco2 cell bioassay. In 30 of the maize genotypes, bioavailable iron increased when degerminated, thus indicating a higher fractional iron uptake because the iron concentrations decreased by more than 70% after the germ fraction was removed. The remaining 22 genotypes showed no change or a decrease in iron bioavailability after degermination. These results confirm previous research showing that the germ fraction is a strong inhibitory component for many maize varieties. Phytate concentrations in maize flours were greatly reduced with degermination. However, the relationship between phytate concentrations and the iron bioavailability of processed maize flour is complex, acting as either inhibitor or promoter of iron uptake depending on the color of the maize kernels and processing method used to produce flour. Other factors in the maize endosperm fractions are likely involved in the effects of degermination on iron bioavailability, such as vitreous or floury endosperm compositions and the polyphenol content of the bran. This study demonstrates that iron nutrition from maize can be enhanced by selecting genotypes where the inhibitory effect of the bran color and endosperm fraction are relatively low, especially after processing *via* degermination.

## Introduction

Severe anemia, primarily due to dietary iron deficiency, affects a large portion of the world’s population, especially among children and women of childbearing age (15 – 49 years) (Stevens et al., 2013). Many regions of the world with a high prevalence of malnutrition rely on staple crops such as wheat, rice and maize (Poole et al., 2021). Typically prepared as flour or meal, maize is the most consumed cereal in Central America, Mexico, and parts of Africa (Ranum et al., 2014). Biofortification of iron in maize is an important strategy in alleviating iron deficiency for populations consuming high-maize diets (Bouis and Welch, 2010). The concentrations of iron in cooked maize are often lower than whole grains, pulses and other cooked vegetables, and iron concentrations in maize are largely dependent on agronomic practices and environment (Rebello et al., 2014; Hindu et al., 2018). Therefore, improving the bioavailability of iron from maize is also an important strategy to ensure more iron is delivered after processing and digestion (Bouis and Welch, 2010; Lung’aho et al., 2011; Glahn et al., 2019).

Significant variation in iron (Fe) bioavailability of maize genotypes has been reported (Oikeh et al., 2003; Lung’aho et al., 2011; Pixley et al., 2011). Using ferritin expression in Caco2 cells as a model of the human intestinal epithelial barrier, Lung’aho et al. (2011) observed a wide range of iron uptake (from 9 to 63 ng ferritin/mg cell protein). Biofortification efforts to improve maize Fe are limited but promising (Oikeh et al., 2003), and target levels for maize Fe and zinc (Zn) have been set with bioavailability taken into consideration (Bouis and Welch, 2010). Although there is a large variation in both Fe concentration and Fe bioavailability among different maize varieties, Fe is more bioavailable from maize when compared to either wheat or rice, which suggests that maize can be a valuable dietary source of iron (Lung’aho et al., 2011; Glahn et al., 2017; Glahn et al., 2019). Furthermore, both *in vitro* and *in vivo* studies have demonstrated that enhanced Fe absorption from maize can be achieved (Lung’aho et al., 2011; Tako et al., 2013). However, the genetic basis for enhanced Fe bioavailability in maize remains unknown (Tako et al., 2015).

Previous research suggests that the germ fraction of maize is the primary inhibitory component for Fe bioavailability (Glahn et al., 2019). Glahn et al. reported that degerminated maize (with the kernel’s germ fraction removed) exhibited higher Fe bioavailability than whole-kernel maize products (Glahn et al., 2019). While the germ fraction contains 27-54% of total Fe, the bioavailability of the germ fraction is low; this effect may be due to high levels of phytate, a prominent iron uptake inhibitor in plant-based foods (Hurrell and Egli, 2010). Glahn et al. also reported that degerminated maize fractions delivered more bioavailable Fe despite being significantly lower in Fe concentration than whole kernels (Glahn et al., 2019). In addition, this study suggested that higher endosperm Fe concentration may yield more bioavailable Fe even in the presence of the germ fraction. Evaluation of wet-milled fractions and degerminated supermarket products further indicated the inhibitory effect of the germ and suggested that degermination can be used to enhance Fe bioavailability from certain maize varieties (Glahn et al., 2019). Given that this previous research only evaluated six maize genotypes, the present study extends this work by evaluating a panel of over 50 maize genotypes to further investigate the role of the germ fraction on maize Fe bioavailability in different kernel types with diverse seed sizes, shapes and colors.

Dietary sources of maize prepared in different regions of the world may or may not contain the germ fraction. While there are several methods to prepare maize, the present study analyzed cooked maize flour (also called instant maize flour) as typically prepared for arepas, a white corn bread commonly consumed in Colombia and Venezuela (Ranum et al., 2014). Producing this type of maize flour normally involves degermination, cooking, steeping, milling, and drying prior to consumer use (Pineda-Gómez et al., 2012). Bioavailability analysis with cooked maize better reflects how iron is absorbed after processing (Glahn et al., 2019).

Several mechanisms could affect Fe bioavailability when maize is processed and degerminated. Phytate is considered a prominent inhibitor of Fe absorption, though correlations between phytate and Fe bioavailability are lacking (Lung’aho et al., 2011) and low-phytate maize has less desirable kernel characteristics (Glahn et al., 2019). Polyphenols, present in the bran (pericarp) of pigmented maize varieties, largely inhibit iron absorption, though some can act as potential enhancers (Hart et al., 2015). Furthermore, differences in the food matrices of flours produced from genotypes with varying bran, germ, and endosperm characteristics may impact Fe bioavailability (Moretti et al., 2006; Zhang and Xu, 2019; Zurak et al., 2020).

This study expands upon existing knowledge related to Fe bioavailability of maize kernel components. The objectives of this study were to evaluate the mineral concentrations (Fe, Zn, Ca and Mg), phytate levels and Fe bioavailability in a diverse panel of cooked whole and degerminated maize genotypes and to further evaluate mechanisms that would improve the Fe bioavailability of this important food crop.

## Materials and methods

### Germplasm materials and sample preparation

Grain from over 50 maize genotypes representing a wide array of phenotypes from different kernel types, improvement levels and colors were sampled for this study (**Table S1**; **Figure S1**). The self-pollinated (inbred line, including public and exPVP) or sibling-pollinated (landrace/heirloom) grain samples were from diverse maize lines grown over several nursery seasons: 2007 Juana Diaz, Puerto Rico; 2013 Columbia, Missouri; 2018 Valle de Banderas, Nayarit, Mexico, and 2019 Columbia, Missouri. Maize samples originated from genotypes grown at a single environment for each nursery season. Ears were harvested after physiological maturity (black layer formation) and dried to approximately 12% moisture before shelling manually to avoid contamination between genotypes. Harvested maize kernels was stored at 4°C at 40% relative humidity until samples were shipped for analysis.

This collection of grain samples was not from plants grown in formal experiments which account for field variability or environmental conditions, but rather were simply chosen to broadly sample maize diversity. In addition, the commercial maize variety Pioneer 3425 (received from the Center for Crops Utilization Research) was used as the reference control for the Caco2 cell culture experiments described below. This variety was selected as a reference standard because it’s commercially available and was extensively studied in previous degermination experiments (Glahn et al., 2019). A total of 52 maize genotypes were grouped together and designated as the Maize Nutrition Panel (MNP). Because environment (locations and years) plays an important role in determining the Fe concentrations of grain, the MNP can be used in future experiments that are designed to control for location and environment variation. A description of the kernel types, improvement levels and colors of the MNP are presented in **Table S1**.

Degerminated samples were produced by carefully splitting each kernel in half, and then removing the germ fraction of each kernel half with a scalpel as previously described by Glahn et al., 2019 (visualized in **Figure S2**). Kernel halves were visually assessed for endosperm composition and recorded as having either vitreous, vitreous and floury, or floury endosperms (**Table S1**; **Figure S1**).

Corn flour samples were prepared based on traditional methods for preparing pre-cooked arepa dough as described by Pineda-Gómez et al. (2012). Whole kernels were cut in half to expose the interior of the kernel in a similar manner as the degerminated grains and yielded similar cooking time across both sample types. Both degerminated and whole maize samples were rinsed in distilled water prior to cooking in 50 mL polyethene centrifuge tubes with five times as much distilled water by weight: approximately 5 g of sample in 25 mL of water. Samples were placed in a tube rack in a pot of cold distilled water on a Max Burton 6400 induction stove and heated from 22°C to 92°C at an average rate of 2.3°C**/**min (visualized in **Figure S3**). The maize was cooked at 92°C for 60 mins, then heat turned off as samples steeped in their cooking water for three hours until they returned to 25°C. Maize samples were drained, weighed, and then frozen at -80°C prior to freeze-drying (VirTis™ Research Equip., Gardiner, NY, United States). Lyophilized samples were weighed again to ensure they achieved at least 50% moisture after cooking. Maize samples that did not achieve 50% moisture after cooking were processed by hand and recooked. Cooked and dried maize samples were milled with a stainless-steel Kinematica Polymix ^®^ analytical hammer mill (PX-MFC 90D, Bohemia, NY, United States) fitted with a 0.5mm screen. These samples represent a pre-cooked maize flour, which is used to prepare arepas and other maize-flour foods.

### Mineral analysis

For mineral analysis, 0.5 g of lyophilized and milled maize sample (in duplicate) from both whole and degerminated grains were predigested in boro-silicate glass tubes with 3 mL of a concentrated ultrapure nitric acid and perchloric acid mixture (60:40 v/v) for 16 h at room temperature. Samples were then placed in a digestion block (Martin Machine) and heated incrementally over 4 h to a temperature of 120°C with refluxing. After incubating at 120°C for 2 h, 2 mL of concentrated ultrapure nitric acid was subsequently added to each sample before raising the digestion block temperature to 145°C for an additional 2 h. The temperature of the digestion block was then raised to 190°C and maintained to evaporate any remaining liquid. Digested samples were resuspended in 20 mL of ultrapure water prior to analysis using ICP-AES (inductively coupled plasma atomic emission spectrometry; Thermo iCAP 6500 Series, Thermo Scientific) with quality control standards (High Purity Standards) following every 10 samples. Yttrium purchased from High Purity Standards (10M67–1) was used as an internal standard. All samples were digested and measured with 0.50 *μ*g/mL of Yttrium (final concentration) to ensure batch-to-batch accuracy and to correct for matrix inference during digestion. All samples were assessed for possible Fe contamination from soil with aluminum (Al) concentrations. None were found to have Al concentrations over 5 μg/g (dry weight), which is the concentration indicative of possible Fe contamination.

### Phytate analysis

For phytate (phytic acid) determination, 0.5 g of each sample (in duplicate) from both whole and degerminated grains was first extracted in 10 mL of 0.66 M hydrochloric acid under constant motion for 16 h at room temperature. A 1 mL aliquot of total extract was collected using a wide bore pipet tip, and then centrifuged at 16,000 g for 10 min to pellet debris. A 0.5 mL sample of supernatant was then neutralized with 0.5 mL 0.75 M sodium hydroxide and stored at -20°C until the day of analysis. A phytate/total phosphorous kit (K-PHYT; Megazyme International, Bray, Ireland) was used to measure liberated phosphorous by phytase and alkaline phosphatase. Phosphorous was quantified by colorimetric analysis as molybdenum blue with phosphorous standards read at a wavelength of 655 nm against the absorbance of a reagent blank. Total phytate concentrations were calculated with Mega-Calc™ by subtracting free phosphate concentrations in the extracts from the total amount of phosphorous that is exclusively released after enzymatic digestion. Phytate to iron molar ratios and phytate to zinc molar ratios are calculated using the following equation (Wiesinger et al., 2022):


$$Phytate\;to\;Fe\;Ratio=\;\left[\frac{\frac{mg}{g}phytate}{660\frac{mg}{mmol}}\right]÷\left[\frac{\frac{mg}{g}iron}{56\frac{mg}{mmol}}\right]$$

$$Phytate\;to\;Zn\;Ratio=\;\left[\frac{\frac{mg}{g}phytate}{660\frac{mg}{mmol}}\right]÷\left[\frac{\frac{mg}{g}zinc}{65\frac{mg}{mmol}}\right]$$

### Caco2 cell bioassay for Fe bioavailability

An established *in vitro* digestion/Caco2 cell culture model of the human intestinal epithelial barrier was used to assess the iron bioavailability of both whole and degerminated maize samples after cooking (Glahn et al., 1998; Glahn, 2022).

Caco2 cells (obtained at passage 21; American Type Culture Collection, Gaithersburg, MD, USA) were seeded at a density of 50,000 cells/cm^2^ in 6-well collagen coated plates (Costar, Cambridge, MA, USA) at passage 28 – 38. Cells were grown for 13 days before each bioassay at 37°C in an incubator with a 5% CO_2_ air atmosphere (constant humidity) using Dulbecco’s modified Eagle’s medium (DMEM; GIBCO, Grand Island, NY) supplemented with 25 mM HEPES (pH 7.2), 10% (v/v) fetal bovine serum (GIBCO) and 1% antibiotic-antimycotic solution (ZellShield^®^, Minerva Biolabs, Germany). The medium was changed every 2 days. Twenty-four hours prior to each bioassay, the culture medium was replaced with iron-free Minimum Essential Medium (MEM [pH 7]; GIBCO) supplemented with 10 mM PIPES (piperazine-N,N’-bis-[2-ethanesulfonic acid]), 1% antibiotic-antimycotic solution, hydrocortisone (4 mg/L), insulin (5 mg/L), selenium (5 μg/L), triiodothyronine (34 μg/L) and epidermal growth factor (20 μg/L). A fresh 1 mL aliquot of MEM (pH 7) covered the cells during each experiment.

A 0.5 g of each sample (in triplicate) of whole and degerminated grain was subjected to a simulated gastric digestion with Porcine pepsin (P6887; 800 – 2500 Units/mg protein; Sigma Aldrich Co., St. Louis, MO, USA) for 1 hour at 37°C (pH 2) and a simulated intestinal digestion with pancreatin (P1750; 4 X USP specifications; Sigma Aldrich) and bile extract (B8631; glycine and taurine conjugates of hyodeoxycholic and other bile salts) for 2 hours at 37°C (pH 7). A sterilized insert ring, fitted with a dialysis membrane, was then inserted into each 6-well plate, thereby creating a two-chamber system. To initiate the bioassay, a 1.5 mL aliquot of the intestinal digest was pipetted into the upper chamber of each well. The plate was covered and incubated at 37°C (5% CO_2_ air atmosphere) on the rocking shaker at 6 oscillations/min for a total of 2 hours. When the bioassay was terminated, the insert ring and digest were removed. The solution in the bottom chamber remained on the cell monolayer and an additional 1 mL of iron-free MEM (pH 7) was added to each well. The cell culture plate was then returned to the incubator (37°C; 5% CO_2_ air atmosphere) for an additional 22 hours, after which the cells were lysed with sterilized water and harvested for analysis.

Ferritin concentration measurements were performed according to the detailed methods described by Glahn et al. (Glahn et al., 1998; Glahn, 2022). The bioassay works according to the following principle: in response to increases in cellular iron concentrations, Caco2 cells produce more ferritin protein, therefore iron bioavailability was determined as the increase in Caco2 cell ferritin production expressed as a ratio to total Caco2 cell protein (ng ferritin per mg of total cell protein) after exposure to a digested sample. Ferritin was measured by enzyme linked immunoassay (Human Ferritin ELISA kit, FRR31-K01, Eagle Biosciences Inc., Nashua, NH, USA) and total cell protein concentrations were quantified using the Bio-Rad DC™ protein assay kit (Bio-Rad Laboratories Inc., Hercules, CA, USA).

To confirm the responsiveness of the Caco2 bioassay, each experiment was run with several quality controls. These include a blank-digest, which is only the physiologically balanced saline and the gastrointestinal enzymes. The blank-digest is used to ensure there is no iron contamination in the bioassay. Ferritin values of Caco2 cells exposed to the blank-digest averaged 2.00 ± 0.54 ng ferritin/mg cell protein (mean ± Standard Deviation; SD) over the course of five cell culture experiments. A cooked, lyophilized, and milled white kidney bean (cultivar name: Snowdon) is run with each assay as a reference standard to index the ferritin/cell protein ratios of the Caco2 cells after being exposed to a digested food matrix. The responsiveness of the Caco2 cells is monitored using a cooked, lyophilized and milled white kidney bean pasta that was processed with the addition of 2 mM ascorbic acid. The maize panel’s reference standard Pioneer 3245 was also monitored before and after degermination. The iron bioavailability of each sample was measured in triplicate.

## Statistical analysis

Statistical analyses and mean separations were determined with SAS 9.4 software (SAS Institute, Cary, NC, USA) using the proc mixed command for the analysis of variance. The normality of residuals for each parameter was evaluated using the Kolmogorov-Smirnov test. Equality of variance for each parameter was determined using the Bartlett’s test. Measured parameters were found to have a normal distribution and equal variance, and were, therefore, acceptable for ANOVA without additional data transformation steps. Genotype was designated as a fixed effect and replication as a random effect followed by a Tukey-Kramer *post-hoc* test. Dot plots illustrating the parameters measured in each maize color type (yellow, white, or pigmented) were developed in GraphPad Prism9 (GraphPad Software, La Jolla, CA, USA). Phenotypic correlations were calculated with Pearson’s 1-tailed correlation test (GraphPad Prism9). Differences with *P* values ≤ 0.05 were considered statistically significant.

## Results

### Maize Fe concentrations and Fe bioavailability vary widely across genotypes

**Figure 1:**
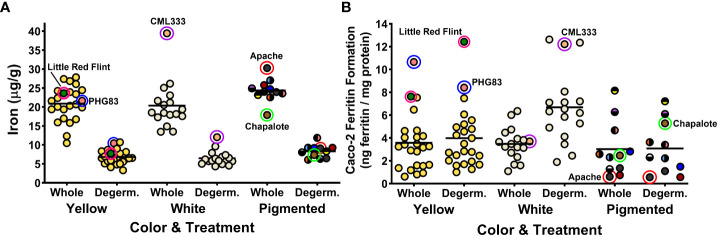
Dot plots depicting iron concentrations **(A)** and iron bioavailability **(B)** of the 52 maize genotypes in the Maize Nutrition Panel before (whole) and after removing the germ fraction (degerminated). Each dot represents the mean value of three measurements from each genotype. Iron concentrations are measured as micrograms per gram of cooked, lyophilized and milled maize sample (dry weight). Iron bioavailability is measured as Caco2 cell ferritin concentrations (ng ferritin/mg total cell protein) after exposure to an *in vitro* digestion of cooked, lyophilized and milled maize sample. Highlighted circles indicate examples of genotypes with unique iron bioavailability traits after degermination. The circle color of pigmented maize represents the bran color pattern of each genotype.

The 52 maize genotypes of the MNP showed a wide range in Fe concentration, Fe bioavailability in whole kernels, and Fe bioavailability in response to degermination. The Fe concentrations of whole cooked maize ranged from 10.5 to 39.5 µg/g while degerminated cooked maize ranged from 3.3 to 12.1 µg/g (**Figure 1A**). Previous reports have found Fe concentrations similar to these ranges (Oikeh et al., 2003; Gwirtz and Garcia-Casal, 2014; Glahn et al., 2019). The Fe bioavailability, measured as ferritin formation by Caco2 cells, ranged from 0.6 to 10.7 ng ferritin/mg cell protein in cooked whole maize and 0.6 to 12.6 ng ferritin/mg cell protein in cooked degerminated maize (**Figure 1B**). Ferritin values for the boiled white kidney bean control averaged 8.13 ± 0.99 ng/mg cell protein (mean ± SD), and the white kidney bean pasta with ascorbic acid control averaged 85.7 ± 13.8 ng/mg cell protein. Ferritin values for the reference standard Pioneer 3245 averaged 2.88 ± 1.15 ng/mg cell protein in whole kernels and 5.18 ± 1.46 ng/mg cell protein in degerminated kernels. A selected group of maize genotypes are highlighted in **Figure 1** to illustrate how the concentrations of Fe are consistently decreased after removing the germ fraction. In contrast, the iron bioavailability response is variable among the highlighted genotypes, with some genotypes having more bioavailable iron after processing (**Figure 1**).

There was no panel-wide correlation between Fe concentrations and the Fe bioavailability of either whole or degerminated maize (**Table 1**). However, both whole and degerminated yellow maize, as well as degerminated white maize showed positive correlations between Fe concentration and Fe bioavailability (**Table 1**). These results indicate that targeted biofortification of Fe into the endosperm fraction of yellow or white maize varieties could be a mechanism by which to enhance the Fe bioavailability of these grain types after processing.

**Table 1:** Pearson correlations and associated *P* values between iron bioavailability, phytate and iron concentrations of the Maize Nutrition Panel (MNP) before and after removing the germ fraction (degerminated)^1^.

	Iron Bioavailability			
	Whole Maize		Degerminated Maize	
Parameter	*Pearson’s* r	*P* value	*Pearson’s* r	*P* value
Entire MNP (n = 52)				
Iron	0.146	0.150	0.175	0.108
Phytate	-0.002	0.496	0.230	0.051
Phytate : Fe Molar Ratio	-0.143	0.155	-0.018	0.437
Yellow (n = 24)				
Iron	0.462	0.012*	0.538	0.003*
Phytate	0.278	0.094	0.461	0.012*
Phytate : Fe Molar Ratio	-0.265	0.105	-0.146	0.248
White (n = 17)				
Iron	0.018	0.473	0.482	0.050*
Phytate	0.044	0.433	0.003	0.496
Phytate : Fe Molar Ratio	0.055	0.418	-0.222	0.196
Pigmented (n = 11)				
Iron	-0.358	0.140	-0.282	0.200
Phytate	-0.583	0.030*	0.258	0.221
Phytate : Fe Molar Ratio	-0.547	0.041*	0.610	0.023*

^1^Correlation coefficients comparing the relationship between iron bioavailability (ng ferritin/mg total cell protein), and the concentrations of phytate (mg/g) and iron (μg/g) in whole or degerminated maize samples after cooking. *P values ≤ 0.05 are consider statistically significant.

### Most but not all genotypes showed an increase in bioavailable Fe when degerminated

For 30 genotypes, degermination resulted in increased Fe bioavailability (**Table 2**). However, 14 genotypes showed no significant change (**Table 3**) and eight had decreased Fe bioavailability after degermination (**Table 4**). White maize genotypes nearly all improved, yellow genotypes varied in response to degermination, and pigmented genotypes largely showed no change or a decrease in Fe bioavailability (**Tables 2**–**4**). Yellow and white maize showed similar average Fe bioavailability in whole cooked maize: 3.6 ± 2.5 ng ferritin/mg cell protein (Mean ± SD) and 3.5 ± 1.4 ng ferritin/mg cell protein, respectively. Group-wide averages of Fe concentrations were similar for yellow and white maize (**Figure 1A**). However, degerminated white maize had higher bioavailable Fe on average (6.7 ng ferritin/mg cell protein) than degerminated yellow maize (4.0 ng ferritin/mg cell protein), which did not show a clear group-wide improvement in bioavailability after processing (**Figure 1B**).

**Table 2:** Description and iron concentrations of maize genotypes that had more bioavailable iron after removing the germ fraction (degerminated)^1^.

		Iron (µg/g)		Caco2 Ferritin (ng/mg cell protein)	
Genotype	Color	Whole	Degerminated	Whole	Degerminated
CML333	White	39.44 ± 1.35 ^a^	12.05 ± 1.55 ^a^	3.69 ± 0.23 ^de^	12.22 ± 0.26 ^a^
Brittain Flint	Yellow	26.84 ± 1.48 ^b^	10.70 ± 1.55 ^ab^	4.17 ± 0.32 ^cd^	5.20 ± 0.25 ^fg^
Andaqui	White	26.16 ± 1.30 ^bc^	9.60 ± 0.07 ^b^	3.48 ± 0.27 ^e^	6.54 ± 1.19 ^cdef^
CML228	Yellow	26.05 ± 0.08 ^b^	9.00 ± 0.15 ^b^	3.47 ± 0.47 ^def^	4.56 ± 0.67 ^gh^
PHT60	White	25.07 ± 0.74 ^cd^	5.69 ± 0.11 ^j^	3.93 ± 0.12 ^cde^	8.06 ± 1.71 ^bcd^
Chapalote	Brown	24.71 ± 0.09 ^c^	9.21 ± 0.36 ^b^	2.47 ± 0.50 ^fg^	5.28 ± 0.51 ^fg^
PA Butter Flavor	White/Yellow/Red	24.00 ± 0.51 ^de^	8.05 ± 0.24 ^cd^	1.27 ± 0.04 ^i^	2.30 ± 0.36 ^jk^
LH54	Yellow	23.93 ± 0.22 ^e^	8.32 ± 0.02 ^c^	4.04 ± 0.49 ^cde^	5.56 ± 0.43 ^efg^
Little Red Flint	Yellow	23.63 ± 0.20 ^e^	7.74 ± 0.33 ^def^	7.63 ± 1.72 ^a^	12.42 ± 0.94 ^a^
Cabuya Amarillo	Orange/Yellow/Red	23.58 ± 0.70 ^ef^	11.87 ± 0.12 ^a^	2.58 ± 0.06 ^g^	3.63 ± 0.24 ^hi^
Oh40B	Yellow	23.39 ± 0.48 ^ef^	5.02 ± 0.02 ^k^	1.60 ± 0.15 ^h^	2.72 ± 0.85 ^ijk^
Harinoso de Ocho	White	23.08 ± 0.21 ^f^	7.91 ± 0.85 ^cdefg^	3.81 ± 0.45 ^cde^	7.12 ± 0.81 ^cd^
Burris Corn	White	21.64 ± 0.32 ^g^	5.80 ± 0.16 ^j^	3.18 ± 0.25 ^ef^	6.20 ± 0.65 ^cde^
Z18-007	White	21.15 ± 0.37 ^g^	6.59 ± 0.48 ^ghi^	4.47 ± 0.60 ^bcd^	5.27 ± 0.70 ^fg^
W64A	Yellow	20.33 ± 0.21 ^h^	4.83 ± 0.08 ^m^	1.55 ± 0.29 ^hi^	2.83 ± 0.86 ^ij^
DE_expt1	Yellow	20.06 ± 0.50 ^hi^	7.74 ± 0.73 ^cdefg^	0.81 ± 0.05 ^j^	1.66 ± 0.06 ^m^
CML52	Yellow	19.40 ± 0.11 ^ij^	6.55 ± 0.20 ^h^	1.37 ± 0.19 ^hi^	1.93 ± 0.13 ^k^
PHW17	White	19.37 ± 0.05 ^j^	5.71 ± 0.20 ^j^	6.01 ± 0.98 ^ab^	12.62 ± 2.94 ^ab^
NC Shoepeg	White	18.54 ± 0.07 ^k^	5.03 ± 0.10 ^km^	1.60 ± 0.53 ^hi^	3.45 ± 0.95 ^hij^
M37W	White	18.44 ± 0.71 ^k^	6.86 ± 0.40 ^fgh^	3.23 ± 0.32 ^ef^	6.33 ± 0.39 ^cde^
Southern Beauty	White	18.14 ± 1.16 ^jkmno^	7.34 ± 0.02 ^e^	1.09 ± 0.18 ^hi^	2.45 ± 0.32 ^jk^
White Gourdseed	White	17.93 ± 0.06 ^m^	4.92 ± 0.02 ^m^	3.66 ± 0.97 ^cdef^	7.21 ± 2.51 ^bcde^
Ahumado	White	17.59 ± 0.38 ^mn^	5.53 ± 0.09 ^j^	3.13 ± 0.16 ^ef^	4.91 ± 0.64 ^fgh^
Olotillo Blanco	White	17.29 ± 0.02 ^no^	6.40 ± 0.04 ^h^	4.74 ± 0.58 ^bc^	7.10 ± 0.76 ^cd^
H100	Yellow	16.98 ± 0.07 ^op^	8.15 ± 0.74 ^cd^	1.18 ± 0.15 ^hi^	2.01 ± 0.04 ^k^
Pioneer 3425	Yellow	15.81 ± 1.18 ^pr^	6.31 ± 0.44 ^hi^	2.91 ± 1.16 ^efg^	4.78 ± 2.06 ^fghi^
CML103	White	14.88 ± 0.21 ^r^	5.83 ± 0.11 ^j^	2.33 ± 0.54 ^fg^	5.84 ± 1.23 ^defg^
CML247	White	13.51 ± 1.07 ^rs^	6.19 ± 0.10 ^i^	6.36 ± 0.71 ^a^	12.35 ± 2.88 ^ab^
CML158Q	White	13.47 ± 0.05 ^s^	4.33 ± 0.36 ^n^	2.71 ± 0.33 ^fg^	4.12 ± 2.07 ^ghi^
LH205	Yellow	12.34 ± 0.46 ^t^	3.32 ± 0.24 °	0.62 ± 0.10 ^k^	1.26 ± 0.49 ^m^

^1^Values are means ± standard deviation of three measurements from each genotype after cooking. Iron concentrations are expressed as micrograms per gram of cooked, lyophilized and milled maize sample (dry weight). Iron bioavailability is expressed as Caco2 cell ferritin formation (ng ferritin/mg total cell protein) after exposure to an in vitro digestion of a cooked, lyophilized and milled maize sample. Mean values sharing the same superscripts in each column are not significantly different at P ≤ 0.05.

**Table 3:** Description and iron concentrations of maize genotypes that had no change in their iron bioavailability after removing the germ fraction (degerminated)^1^.

		Iron (µg/g)		Caco2 Ferritin (ng/mg cell protein)	
Genotype	Color	Whole	Degerminated	Whole	Degerminated
Apache	Black	30.27 ± 0.37 ^a^	9.08 ± 0.63 ^a^	0.60 ± 0.06 ^h^	0.56 ± 0.05 ^h^
Pa875	Yellow	27.84 ± 0.06 ^b^	8.69 ± 0.48 ^a^	4.66 ± 0.69 ^c^	4.94 ± 0.99 ^b^
LH57	Yellow	27.38 ± 0.39 ^bc^	7.46 ± 0.18 ^b^	6.48 ± 0.75 ^b^	7.48 ± 1.01 ^a^
Ancho King	White/Blue	27.03 ± 0.32 ^c^	9.26 ± 0.25 ^a^	2.31 ± 0.10 ^e^	2.22 ± 0.36 ^d^
Lail Flint	White/Yellow/Blue	24.92 ± 0.52 ^d^	6.33 ± 0.10 ^d^	6.27 ± 0.80 ^b^	6.10 ± 0.71 ^ab^
Maiz Morado	Black	23.94 ± 0.75 ^d^	6.45 ± 0.05 ^cd^	1.36 ± 0.15 ^f^	1.10 ± 0.30 ^fg^
Cariaco-Costeno	Yellow/Pink	22.55 ± 0.03 ^e^	6.10 ± 0.55 ^de^	8.15 ± 1.44 ^a^	7.21 ± 0.24 ^a^
John Haulk	Yellow	21.60 ± 0.14 ^f^	6.99 ± 0.02 ^c^	7.54 ± 1.29 ^ab^	6.06 ± 0.60 ^a^
Jackie Freeman	Yellow	20.70 ± 0.44 ^g^	5.92 ± 0.28 ^e^	2.81 ± 0.35 ^de^	2.77 ± 1.16 ^cde^
Cherokee Flour	White	20.56 ± 0.47 ^gh^	4.56 ± 0.44 ^g^	1.67 ± 0.19 ^f^	1.89 ± 0.07 ^de^
A619	Yellow	19.88 ± 0.34 ^ghi^	5.74 ± 0.07 ^e^	1.65 ± 0.17 ^f^	1.58 ± 0.19 ^ef^
PHM49	Yellow	19.36 ± 0.56 ^i^	5.10 ± 0.06 ^f^	3.24 ± 0.10 ^d^	3.49 ± 0.13 ^c^
Mo47	Yellow	15.93 ± 0.12 ^j^	5.54 ± 0.07 ^e^	4.07 ± 0.18 ^c^	4.24 ± 0.39 ^b^
Tx303	Yellow	10.47 ± 0.04 ^k^	4.10 ± 0.04 ^g^	0.92 ± 0.03 ^g^	1.00 ± 0.06 ^g^

^1^Values are means ± standard deviation of three measurements from each genotype after cooking. Iron concentrations are expressed as micrograms per gram of cooked, lyophilized and milled maize sample (dry weight). Iron bioavailability is expressed as Caco2 cell ferritin formation (ng ferritin/mg total cell protein) after exposure to an in vitro digestion of a cooked, lyophilized and milled maize sample. Mean values sharing the same superscripts in each column are not significantly different at P ≤ 0.05.

**Table 4:** Description and iron concentrations of maize genotypes that had less bioavailable iron after removing the germ fraction (degerminated)^1^.

		Iron (µg/g)		Caco2 Ferritin (ng/mg cell protein)	
Genotype	Color	Whole	Degerminated	Whole	Degerminated
Jimmy Red	Red	25.78 ± 0.11 ^a^	9.27 ± 0.37 ^a^	0.74 ± 0.06 ^f^	0.57 ± 0.02 ^e^
MBST	Yellow	24.45 ± 0.11 ^b^	6.51 ± 0.03 ^d^	3.27 ± 1.39 ^bcd^	1.70 ± 0.37 ^d^
S8326	Yellow	24.25 ± 0.05 ^b^	5.99 ± 0.01 ^e^	3.41 ± 0.25 ^c^	2.49 ± 0.39 ^c^
Kulli	White/Yellow/Red	23.17 ± 0.69 ^c^	8.32 ± 0.36 ^b^	4.67 ± 0.87 ^b^	3.39 ± 0.22 ^b^
CHZM 05 003	Yellow	22.36 ± 0.02 ^c^	6.49 ± 0.12 ^d^	4.62 ± 0.14 ^b^	3.99 ± 0.30 ^b^
PHG83	Yellow	21.65 ± 0.21 d	10.44 ± 0.83 ^a^	10.65 ± 1.28 ^a^	8.41 ± 0.47 ^a^
Ohio Blue Clarage	Blue	17.89 ± 0.33 ^e^	7.36 ± 0.71 ^bc^	2.81 ± 0.41 ^d^	1.49 ± 0.16 ^d^
LH196	Yellow	16.75 ± 0.44 ^f^	6.71 ± 0.51 ^cd^	3.16 ± 0.40 ^cd^	2.45 ± 0.25 ^c^

^1^Values are means ± standard deviation of three measurements from each genotype after cooking. Iron concentrations are expressed as micrograms per gram of cooked, lyophilized and milled maize sample (dry weight). Iron bioavailability is expressed as Caco2 cell ferritin formation (ng ferritin/mg total cell protein) after exposure to an in vitro digestion of a cooked, lyophilized and milled maize sample. Mean values sharing the same superscripts in each column are not significantly different at P ≤ 0.05.

### White maize showed the largest increase in Bioavailable Fe when degerminated

All white maize genotypes except for Cherokee Flour had improved Fe bioavailability when degerminated. Bioavailable Fe ranged from 1.1 to 6.4 ng ferritin/mg cell protein in whole white maize and from 3.4 to 12.6 ng ferritin/mg cell protein when degerminated. While no whole-kernel white maize exceeded 7 ng ferritin/mg cell protein, three genotypes achieved over 12 ng ferritin/mg cell protein after degermination (**Figure 1B**). The bioavailable Fe of PHW17 and CML247 approximately doubled when degerminated while that of CML333 more than tripled (**Figure 1B**; **Table 2**). Only Cherokee flour, with low bioavailable Fe (1.67 ± 0.19 ng ferritin/mg cell protein) when whole, showed no change when degerminated (**Table 3**). There was no significant correlation between cooked Fe concentrations and the Fe bioavailability of whole white maize, however, there was a positive correlation between Fe concentrations and Fe bioavailability when degerminated (**Table 1**). These results suggest that the germ fraction is a strong inhibitor of iron bioavailability in white maize, and breeding for higher endosperm Fe concentrations could be an affective strategy to enhance the absorption of iron from white maize after processing.

### Yellow Maize Genotypes Showed Mixed Responses to Degermination

Yellow maize showed mixed results in terms of degermination and Fe bioavailability: 11 genotypes improved, eight had no change, and five had less bioavailable Fe after degermination (**Tables 2**–**4**). Little Red Flint was among the highest in Fe bioavailability of the whole-kernel yellow genotypes (7.74 ± 0.33 ng ferritin/mg cell protein) and its Fe bioavailability increased (12.42 ± 0.94 ng ferritin/mg protein) when degerminated (**Figure 1B**; **Table 2**). In contrast, while PHG83 had the highest Fe bioavailability (10.65 ± 1.28 ng ferritin/mg cell protein) of the yellow whole-kernel genotypes, its Fe bioavailability decreased (8.41 ± 0.47 ng ferritin/mg cell protein) after processing (**Figure 1B**; **Table 4**). Bioavailable Fe ranged from 0.8 to 10.7 ng ferritin/mg cell protein in whole-kernel samples, and 1.0 to 12.4 ng/mg total protein in degerminated samples. There was a significant correlation between cooked Fe concentrations and the Fe bioavailability of yellow maize before and after degermination (**Table 1**). These results indicate that breeding for overall higher iron concentrations could lead to more bioavailable iron from yellow maize after processing.

### Pigmented maize has less bioavailable Fe, which does not improve after degermination

When compared to white and yellow maize, pigmented genotypes generally had less bioavailable Fe including mixed-color samples (**Figure 1**; **Tables 3**, **4**). These genotypes showed either no change or a decrease in bioavailable Fe after degermination except for Chapalote (brown), which increased from 2.47 ± 0.50 to 5.28 ± 0.51 ng ferritin/mg cell protein (**Figure 1B**; **Table 2**). Black pigmented genotypes Apache and Maiz Morado did not show a significant change (**Table 3**), while red genotype Jimmy Red and blue genotype Ohio Blue Clarage had a decrease in Fe bioavailability after processing (**Table 4**). There was no significant correlation between Fe concentrations and the Fe bioavailability of pigmented maize before or after degermination (**Table 1**).

The blue, red, purple, black, or brown bran of pigmented genotypes (**Figure S1**) indicate the presence of polyphenols. The types of polyphenols present in the bran of maize vary greatly between genotypes (Suriano et al., 2021). Polyphenols are largely considered inhibitors of iron absorption, however, the composition of individual polyphenols that create the different bran colors may complicate this assertion: while many types of polyphenols inhibit iron absorption, some can act as enhancers (Hart et al., 2015). Overall, the lack of Fe bioavailability in pigmented maize, both whole and degerminated, is likely due to the presence of inhibitory polyphenols such as tannins and anthocyanins in the bran of blue, red, purple, and black maize.

### The relationship between phytate and the Fe bioavailability of maize is complex

**Figure 2:**
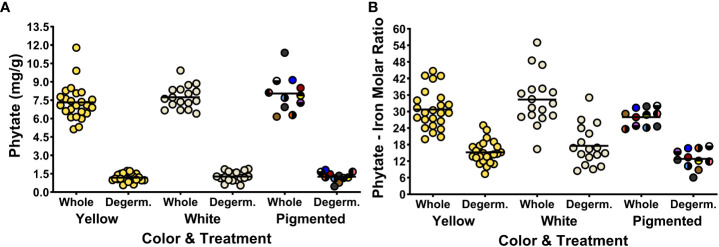
Dot plots depicting phytate concentrations **(A)** and phytate:Fe molar ratios **(B)** of the 52 maize genotypes in the Maize Nutrition Panel before (whole) and after removing the germ fraction (degerminated). Each dot represents the mean value of two measurements from each genotype. Phytate concentrations are measured as milligrams per gram of cooked, lyophilized and milled maize sample (dry weight). The circle color of pigmented maize represents the bran color pattern of each genotype.

**Figure 3:**
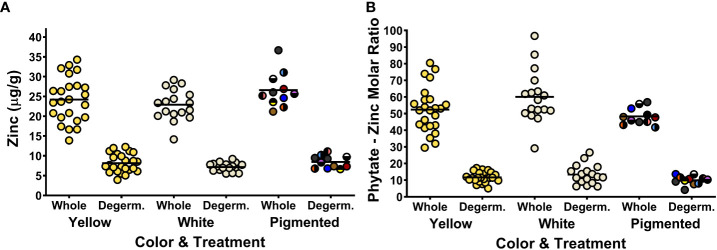
Dot plots depicting zinc concentrations **(A)** and phytate:Zn molar ratios **(B)** of the 52 maize genotypes in the Maize Nutrition Panel before (whole) and after removing the germ fraction (degerminated). Each dot represents the mean value of two measurements from each genotype. Zinc concentrations are measured as micrograms per gram of cooked, lyophilized and milled maize sample (dry weight). The circle color of pigmented maize represents the bran color pattern of each genotype.

**Figure 2** shows the phytate concentrations and phytate:Fe molar ratios of cooked maize samples before and after degermination. The phytate concentration and the phytate:Fe molar ratio of each genotype in the MNP are listed in **Table S2**. Removing the germ fraction reduced the phytate concentrations by sixfold (from a panel-wide average of 7.6 ± 1.3 to 1.2 ± 0.4 mg/g) and halved the average phytate:Fe molar ratios, from 31.1 ± 7.9 to 15.5 ± 5.4 (**Figure 2**; **Table S2**). Overall, phytate and Fe concentrations were positively correlated among whole and degeminated maize genotypes in the MNP and for all color groups besides white maize (**Table 5**). The loss of phytate, Fe and Zn after removing the germ fraction indicates that these compounds and elements are colocalized within the germ fraction (**Figures 2**, **3**; **Tables S2-S3**). However, the large reductions in phytate:Fe and phytate:Zn molar ratios after degermination reveal that Fe and Zn may have potentially localized with other compounds in addition to phytate in the bran and endosperm fraction of the maize kernel (**Figures 2**, **3**; **Table S2**).

**Table 5:** Pearson correlations and associated *P* values between iron and the concentrations of zinc and phytate in the Maize Nutrition Panel (MNP) before and after removing the germ fraction (degerminated)^1^.

	Iron			
	Whole Maize		Degerminated Maize	
Parameter	*Pearson’s* r	*P* value	*Pearson’s* r	*P* value
Entire MNP (n = 52)				
Zinc	0.591	<0.0001*	0.357	0.005*
Phytate	0.328	0.009*	0.390	0.002*
Phytate : Fe Molar Ratio	-0.724	<0.0001*	-0.569	<0.0001*
Yellow (n = 24)				
Zinc	0.744	<0.0001*	0.382	0.033*
Phytate	0.385	0.032*	0.597	0.001*
Phytate : Fe Molar Ratio	-0.710	<0.0001*	-0.487	0.008*
White (n = 17)				
Zinc	0.248	0.169	-0.219	0.199
Phytate	0.019	0.471	0.114	0.332
Phytate : Fe Molar Ratio	-0.806	<0.0001*	-0.467	0.029*
Pigmented (n = 11)				
Zinc	0.880	0.0002*	0.445	0.085
Phytate	0.775	0.003*	0.622	0.021*
Phytate : Fe Molar Ratio	0.105	0.380	-0.013	0.484

^1^Correlation coefficients comparing the relationship between iron concentrations (μg/g), and the concentrations of zinc (μg/g) and phytate (mg/g) and in whole or degerminated maize samples after cooking. *P values ≤ 0.05 are consider statistically significant.

The results in **Table 1** show no correlation between Fe bioavailability and phytate concentrations or phytate:Fe molar ratios among the 52 genotypes of the MNP when prepared with either whole kernels or degerminated flour. Moreover, there was no correlation between Fe bioavailability and phytate in white and yellow maize flour prepared with whole kernels. In contrast, there was strong negative correlation between Fe bioavailability and phytate concentrations in pigmented maize flour prepared with whole kernels. However, this correlation evidently did not translate to more bioavailable Fe when compared to the white and yellow maize types. The lack of any consistent correlation between the Fe bioavailability, phytate concentrations or phytate:Fe molar ratios of genotypes in the MNP indicates that phytate concentrations of whole kernel maize flour may not be as meaningful for maize Fe bioavailability as previously suggested (Hurrell and Egli, 2010; Lung’aho et al., 2011; Glahn et al., 2019).

The relationship between Fe bioavailability and phytate becomes more dynamic when degerminated maize is prepared into a cooked flour product. Interestingly, there was a strong positive correlation between Fe bioavailability and concentrations of phytate in yellow maize after degermination (**Table 1**). There was also a strong positive correlation between Fe bioavailability and phytate:Fe molar ratios among the pigmented maize genotypes (**Table 1**). A fully intact phytate molecule (IP6) is often considered an inhibitor of dietary iron uptake, but is susceptible to heat and processing. Boiling, steeping and milling maize into flour contributes to the degradation of phytate into smaller molecules (IP5, IP4, IP3, etc.) with less inhibitory actions. Phytate degradation after processing degerminated maize flour could change the phytate-Fe interactions within endosperm, allowing for greater iron uptake during digestion (Wiesinger et al., 2022; Gupta et al., 2015).

### Zinc, calcium and magnesium concentrations of the MNP

The concentrations of Zn, Ca and Mg for the 52 genotypes in the MNP - before and after degermination - are listed in **Tables S3-S4**. **Figure 3A** shows the concentrations of zinc in whole and dergerminated maize samples after cooking. Large reductions in Zn concentrations were measured in all the genotypes after degermination (**Figure 3A**). Phytate : Zn molar ratios were also significantly lower after removing the germ fraction, with values nearly halve of whole maize kernels (**Figure 3B**). Phytate : Zn molar ratios are considered a proxy for estimating zinc bioavailability from foods (Wiesinger et al, 2022). How the decrease in phytate:Zn molar ratios impact the bioavailability of Zn from processed maize remains unknown, but as demonstrated with Fe, increasing the Zn concentrations in the endosperm fraction rather than the germ fraction could be a strategy to improve the delivery of bioavailable Zn from degerminated maize flour.

The concentrations of Fe and Zn had significant positive correlations for all sample groups except for white maize and degerminated pigmented samples (**Table 5**). Previous research has also reported a positive correlation between Fe and Zn concentrations for some, but not all maize sample groups (Pixley et al., 2011). These data suggest that selection for higher Fe concentrations, especially in yellow maize, may inherently lead to higher Zn concentrations as well (**Table 5**).

## Discussion

This study expanded upon previous maize Fe bioavailability research (Glahn et al., 2019) and found a wide range in Fe bioavailability across a large number of genotypes both in whole and degerminated kernels. There may be increased bioavailable Fe genotypes already available for research and improvement: degerminated white CML333 and orange Little Red Flint had twice as much bioavailable Fe than the average for their kernel color group (**Figure 1**). Other genotypes including yellow PHG83 may be good candidates for increased bioavailable Fe in whole kernel maize preparations. These and other genotypes in this study with higher-than-average bioavailable Fe may be candidates for the development of high-bioavailable Fe varieties. This study clearly indicates that additional diversity panels and quantitative trait locus (QTL) mapping populations should be developed and utilized to reach the achievable goal of producing more high bioavailable Fe maize varieties. Indeed, a recombinant inbred line (RIL) population of B73 x CML333 is a ready-made resource, as CML333 is one of the nested association mapping founders (McMullen et al., 2009). Because the population has already been genotyped, QTL mapping would only require phenotyping of the ~200 RILs followed by QTL analysis. Unfortunately, Little Red Flint is a landrace/heirloom variety with no current genetic resources. It would be interesting to create a population from the cross of CML333 and Little Red Flint to see if these two varieties have different mechanisms/genes leading to their higher bioavailable Fe and/or whether transgressive segregation could result in progeny higher than either of the parents. Producing this population in multiple locations over several growing seasons would also be an important step in monitoring the variability of iron bioavailability among RILs in different environments.

The amount of bioavailable Fe in some maize genotypes can match or even exceed that of crops typically considered good sources of iron, such as white beans. Two of the MNP whole-kernel samples and six of the degerminated samples matched or exceeded the iron bioavailability of a cooked white kidney bean (*cv*. Snowdon) considered to be high in bioavailable Fe. Ferritin values for the white kidney bean averaged 8 ng ferritin/mg cell protein. Although maize typically has lower Fe concentrations than grain legumes (Glahn et al., 2019), its chemical composition and food matrix has the potential to deliver more dietary iron due to its high bioavailability during digestion.

Increasing iron absorption from maize *via* genotype selection and or processing could improve the iron status of vulnerable populations in places where maize is a major part of the diet. For example, if iron absorption were doubled in maize consumed in Tanzania, where more than half of the diet is maize in some regions (Cochrane and D’Souza, 2015), the rates of iron deficiency – which affects over 40% of the women and children (National Bureau of Statistics (NBS), 2011) – could be decreased.

While previous research suggested that degermination consistently increases bioavailability (Glahn et al., 2019), the current study indicates that while Fe bioavailability from many genotypes may benefit from degermination, several show no change or even a decrease in Fe bioavailability. Most of the white and yellow maize genotypes increased in bioavailable Fe when degerminated, indicating lower concentrations of polyphenols may be beneficial in improving the absorption of iron from maize after processing. In addition, it should also be considered that degerminated maize may have less of an inhibitory effect than whole maize on other sources of Fe consumed in the same meal, such as Fe from beans or other whole grains. The approach of using a Caco2 cell bioassay is ideally suited to such studies (Glahn et al., 2017) as it is a model of the human intestinal epithelial barrier.

White maize is typically preferred for human consumption (Gwirtz and Garcia-Casal, 2014; Ranum et al., 2014). Arepas, and the cooked maize flour precursor as prepared in this study, are typically made with white corn (Pineda-Gómez et al., 2012). While there are nutritional issues with consuming white maize, such as the lack of vitamin A precursors (Ranum et al., 2014), the benefits of increased iron accessibility in degerminated white maize flour suggests that white maize a promising candidate for iron biofortification. In the present study white maize almost always increased in Fe bioavailability when degerminated and three of the four highest-bioavailable Fe samples in the MNP were degerminated white maize (**Figure 1**). The lack of iron inhibiting polyphenols in the bran is most likely why Fe bioavailability responds more positively to degermination in white maize than other color groups (Hart et al., 2015).

In yellow maize, differences between genotypes should be considered when determining whether degermination will improve Fe bioavailability. Yellow maize contains more carotenoids than white maize, which have been reported to enhance iron absorption even in the presence of inhibitors such as tannins and phytate (García-Casal et al., 2000; Pixley et al., 2011). However, this study shows that yellow maize had similar Fe bioavailability to white maize in whole kernels (**Figure 1**). Although carotenoids were not measured, the results of this study indicate there is no clear alignment between the yellow color of maize and Fe bioavailability.

Alternatively, this research demonstrates a positive relationship between Fe and Zn concentrations in yellow maize (**Table 5**). Biofortification of zinc in maize is an important strategy aimed at alleviating micronutrient deficiencies in the same regions where maize is a dietary staple (Gallego-Castillo et al., 2021). High kernel-zinc maize varieties are available to consumers in Latin American, and can deliver more dietary zinc when prepared into either arepas, tortillas or mazamorra (Gallego-Castillo et al., 2021). A negative correlation between Zn and Fe bioavailability in maize has been previously reported but was not consistent across all trials (Pixley et al., 2011). Zn to Fe concentration ratios were 0.9 ± 0.2 and 0.9 ± 0.3 for whole and degerminated maize samples, respectively. These values are far lower than the 5 to 1 ratio suggested as the threshold for Zn inhibition of Fe absorption (Olivares et al., 2012).

The methods by which maize is processed into food affects Fe bioavailability, as the process of degermination evaluated in this study clearly shows. Some traditional and industrial maize processing methods remove the germ fraction while others retain it. Degermination is desirable because it extends the shelf life of maize flour (Gwirtz and Garcia-Casal, 2014). Production of pre-cooked corn flours for arepas – the process used in this study – usually includes a degermination step, while nixtamalization for tortillas does not (Pineda-Gómez et al., 2012). Further common maize processing methods, such as soaking and fermentation vary in their capacity to degrade phytate, but have less of an effect on iron absorption (Hurrell and Egli, 2010; Gupta et al., 2015). Variation of Fe concentrations in different white maize preparations has been previously reported: 24 µg/g Fe in whole-grain maize flour, 9 µg/g in degermed flour, 15 µg/g nixtamalized dough, and 9 µg/g in precooked maize flour (Gwirtz and Garcia-Casal, 2014). The bioavailability of Fe across aspects of processing beyond degermination likely varies as well, with genotype playing a role in these differences. Local preferences for maize processing methods and maize color groups should be considered when developing high bioavailable Fe maize (Bouis and Welch, 2010).

The matrix of a food clearly impacts iron absorption, but these effects are not well understood (Moretti et al., 2006). Ingredients that are served with maize may either inhibit or enhance Fe bioavailability (Bouis and Welch, 2010). Ingredients that include ascorbic acid, for example, can significantly increase the amount of iron absorbed from a meal (Hurrell and Egli, 2010). Maize is frequently consumed in dishes with the common bean, which can also contain high levels of phytate and polyphenols, both of which reduce iron absorption (Petry et al., 2010; Wiesinger et al., 2020). In maize itself, there are certain fibers present that have been reported to reduce Fe and Zn bioavailability (Bouis and Welch, 2010). The characteristics of the endosperm and germ matrices likely affect the accessibility of iron in maize. The maize endosperm can be described on a range of floury to vitreous. Vitreous and floury endosperms differ in zein, amylose, starch, and lipid compositions and differ in a range of physicochemical properties (Zhang and Xu, 2019; Zurak et al., 2020). Zeins may be relevant to iron absorption because specific zein proteins (gamma and beta) are rich in cysteine (Wu et al., 2012). Cysteine and cysteine-containing peptides have been found to enhance iron absorption (Hurrell and Egli, 2010). In the present study, three of the four genotypes with the highest bioavailable Fe had among the most vitreous endosperms – exceeding ferritin values of 12 ng ferritin/mg cell protein when degerminated (**Table S1**). Interestingly, the maize genotype with the highest iron bioavailability (PHW17) had a mixture of vitreous and floury endosperm (**Figure S1**; **Table S1**). There were no obvious trends in whole kernels, possibly related to the more prominent effects of the germ fraction. However, this study did not quantify the vitreous:floury endosperm ratio or statistically analyze any correlations between endosperm composition and bioavailable Fe.

## Conclusions

This study reveals that there are ways to improve the iron bioavailability of maize *via* breeding and processing. Indeed, high bioavailable Fe maize is achievable (Tako et al., 2013; Tako et al., 2015). The common practice of degermination clearly alters the iron delivery from maize, often increasing iron bioavailability, though effects vary by kernel type, endosperm characteristics and bran color. This research provides new insights into maize as an improved dietary source of Fe, demonstrating that the phenotype for high Fe bioavailability after processing is an important factor to consider when breeding for the biofortification of maize. Furthermore, processes that confer increased Fe absorption may already be available for maize. Implementing these ideas to increase the iron bioavailability of maize in food products of convenience (i.e., snacks, pasta, and baked goods) is an additional strategy to help lower the prevalence of iron deficiency anemia in regions where cereal grains make up a high proportion of a communities’ diet. Measuring the iron content and iron bioavailability of maize products from multiple growing environments over several years of experiments will be the next step in evaluating the iron benefits of maize, especially in genotypes that show a high capacity for iron bioavailability after cooking and processing.

## Data availability statement

The original contributions presented in the study are included in the article/**Supplementary Material**. Further inquiries can be directed to the corresponding author.

## Author contributions

JK: conceptualization and writing – original draft. JK, JW, and SF-G: investigation and methodology. JK and JW – data curation and formal analysis. SF-G and RG - funding acquisition, project administration, and resources. JK, JW, SF-G, and RG – writing – review and editing. All authors contributed to the article and approved the submitted version.
