# Supplementary material for: Iron bioavailability of maize (Zea mays L.) after removing the germ fraction
Source: Front Plant Sci. 2023 Mar 7;14:1114760. doi: 10.3389/fpls.2023.1114760 (PMC10029919; doi:10.3389/fpls.2023.1114760)
Supplement: Supplementary file 1 [file DataSheet_1.pdf]

Supplementary Table S1. Color, improvement level and endosperm characteristics of the fifty-two maize genotypes selected for nutritional evaluation before and after degermination.

Genotype	Color[1]	Improvement Level [2]	Endosperm
Pioneer 3245	Yellow (O-Y)	hybrid	Vitreous & Floury
LH54	Yellow (O-Y)	inbred, exPVP	Floury
LH57	Yellow (O-Y)	inbred, exPVP	Floury
LH196	Yellow (O-Y)	inbred, exPVP	Floury
LH205	Yellow (O-Y)	inbred, exPVP	Floury
PHG83	Yellow (O-Y)	inbred, exPVP	Floury
PHM49	Yellow (O-Y)	inbred, exPVP	Floury
MBST	Yellow (O-Y)	inbred, exPVP	Vitreous & Floury
S8326	Yellow (O-R)	inbred, exPVP	Vitreous & Floury
W64A	Yellow (BY)	inbred, temperate	Floury
Mo47	Yellow (BY)	inbred, temperate	Floury
H100	Yellow (O-Y)	inbred, temperate	Vitreous & Floury
A619	Yellow (BY)	inbred, temperate	Floury
Pa875	Yellow (BY)	inbred, temperate	Floury
DE_expt1	Yellow (O-Y)	inbred, temperate	Floury
Oh40B	Yellow (Dark O-R)	inbred, temperate	Vitreous & Floury
CML52	Yellow (O-Y)	inbred, tropical	Vitreous & Floury
Tx303	Yellow (O-Y)	inbred, tropical	Floury
CML228	Yellow (O-Y)	inbred, tropical	Vitreous
CHZM 05 003	Yellow (BY)	landrace/heirloom	Floury
Little Red Flint	Yellow (O-Y)	landrace/heirloom	Vitreous
John Haulk	Yellow (O-Y)	landrace/heirloom	Vitreous
Jackie Freeman	Yellow (Dark O-R)	landrace/heirloom	Vitreous & Floury
Brittain Flint	Yellow (O-Y)	landrace/heirloom	Vitreous
PHW17	White	inbred, exPVP	Vitreous & Floury
PHT60	White	inbred, exPVP	Vitreous & Floury
CML103	White	inbred, tropical	Vitreous & Floury
CML247	White	inbred, tropical	Vitreous
CML333	White	inbred, tropical	Vitreous
M37W	White	inbred, tropical	Vitreous & Floury
CML158Q	White	inbred, tropical	Vitreous & Floury
Olotillo Blanco	White	landrace/heirloom	Vitreous & Floury
Ahumado	White	landrace/heirloom	Vitreous & Floury
Z18-007	White	landrace/heirloom	Vitreous & Floury
Andaqui	White	landrace/heirloom	Vitreous & Floury
Cherokee Flour	White	landrace/heirloom	Floury
Harinoso de Ocho	White	landrace/heirloom	Floury
White Gourdseed	White	landrace/heirloom	Vitreous & Floury
Burris	White	landrace/heirloom	Vitreous & Floury
Southern Beauty	White	landrace/heirloom	Vitreous & Floury
NC Shoepeg	White	landrace/heirloom	Vitreous
Jimmy Red	Red	landrace/heirloom	Vitreous & Floury
Chapalote	Brown	landrace/heirloom	Vitreous
Ohio Blue Clarage	Blue	landrace/heirloom	Vitreous & Floury
Maiz Morado	Black-Purple	landrace/heirloom	Floury
Apache	Black-Red	landrace/heirloom	Floury
Cabuya Amarillo	Mix (Y, O-R)	landrace/heirloom	Floury
Cariaco-Costeno	Mix (Y, P-Y)	landrace/heirloom	Floury
Kulli	Mix (W, Y, P-O, R)	landrace/heirloom	Floury
PA Butter Flavor	Mix (W, R-W, P)	landrace/heirloom	Vitreous
Ancho King	Mix (B, W)	landrace/heirloom	Vitreous & Floury
Lail Flint	Mix (W, Y, Y-O, R, B, B-R)	landrace/heirloom	Floury

[1] Color Patterns: B, Blue; BY, Bright Yellow; B-R, Blue-Red Striped; O-R, Orange-Red; O-Y, Orange-Yellow; P-O, Pink-Orange; P, Purple; P-Y, Pink-Yellow; R-W, Red-White Striped; W, White; Y, Yellow; Y-O, Yellow-Orange Striped. [2] Improvement Level: inbreds were divided into ex-PVP (expired Plant Variety Protection; private industry; generally temperate lines), temperate (public), and tropical (public). Landraces are unimproved populations equivalent to heirlooms in other crops.

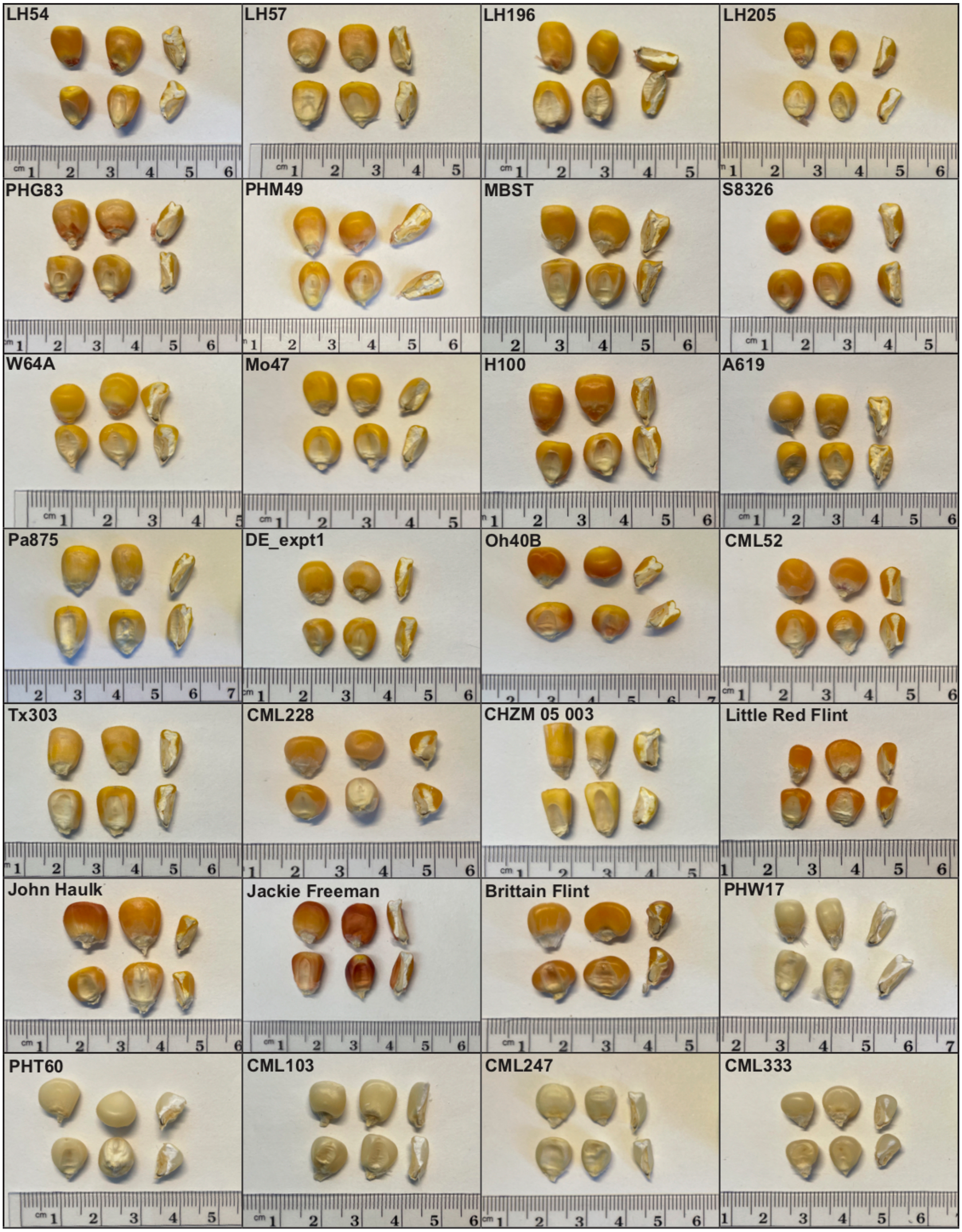

LH54
LH57
LH196
LH205
PHG83
PHM49
MBST
S8326
W64A
Mo47
H100
A619
Pa875
DE_expt1
Oh40B
CML52
Tx303
CML228
CHZM 05 003
Little Red Flint
John Haulk
Jackie Freeman
Brittain Flint
PHW17
PHT60
CML103
CML247
CML333

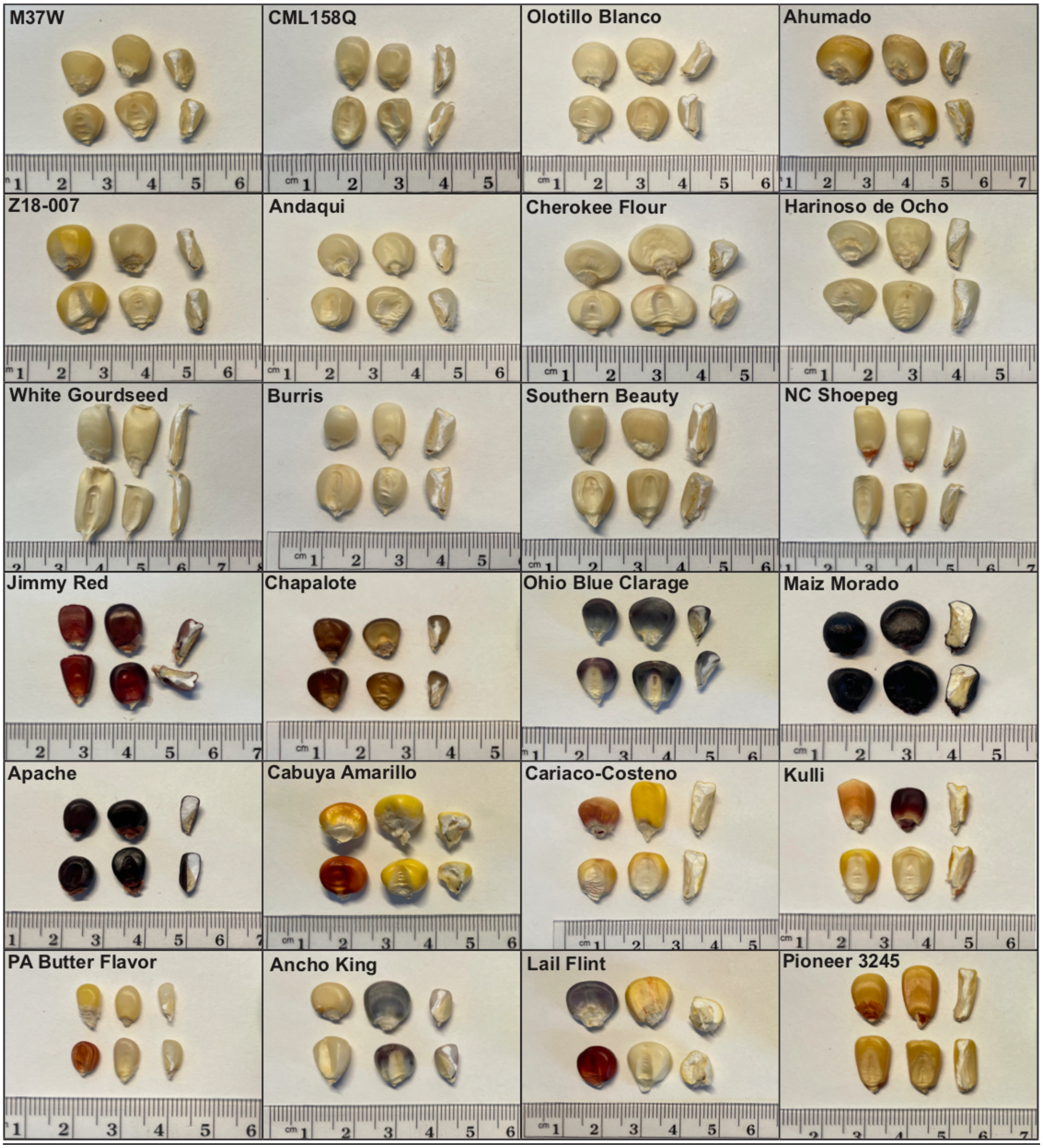

Supplementary Figure S1. Fifty-two maize genotypes organized by color. Pioneer 3245 was used as reference standard when evaluating the iron bioavailability of this panel. Kernels on the right-hand side are cut in half to show composition of either vitreous (beige or pigmented) or floury (white) endosperm. Scale is shown in cm.

Supplementary Table S2. Phytate concentrations and phytate to iron molar ratios of the Maize Nutrition Panel before and after degermination.[1]

		Phytate (mg/g)		Phytate - Iron Molar Ratio	
Genotype	Color	Whole	Degerminated	Whole	Degerminated
Pioneer 3245	Yellow	5.13 ± 0.73	1.05 ± 0.57	27.5 ± 4.7	14.2 ± 1.6
LH54	Yellow	6.77 ± 0.77	1.73 ± 0.09	24.0 ± 2.5	17.6 ± 1.0
LH57	Yellow	7.61 ± 1.41	0.97 ± 0.04	23.6 ± 4.7	11.0 ± 0.7
LH196	Yellow	6.41 ± 1.33	1.51 ± 0.36	32.5 ± 5.9	19.0 ± 14.1
LH205	Yellow	6.10 ± 0.60	0.98 ± 0.70	41.9 ± 5.7	25.0 ± 19.7
PHG83	Yellow	7.09 ± 0.37	1.49 ± 0.16	27.8 ± 1.7	12.1 ± 2.2
PHM49	Yellow	7.76 ± 0.02	1.40 ± 0.29	34.0 ± 1.1	23.3 ± 4.5
MBST	Yellow	5.98 ± 2.93	0.57 ± 0.13	20.8 ± 10.2	7.36 ± 1.66
S8326	Yellow	7.09 ± 3.00	1.05 ± 0.84	24.8 ± 10.6	14.9 ± 11.8
W64A	Yellow	7.08 ± 0.20	0.62 ± 0.10	29.5 ± 0.5	10.9 ± 1.6
Mo47	Yellow	8.06 ± 0.02	0.99 ± 0.69	42.9 ± 0.3	15.2 ± 10.6
H100	Yellow	7.43 ± 1.87	0.96 ± 0.23	37.1 ± 9.5	10.0 ± 1.5
A619	Yellow	6.90 ± 1.04	1.39 ± 0.11	29.4 ± 4.9	20.5 ± 1.8
Pa875	Yellow	6.56 ± 0.27	1.17 ± 0.47	20.0 ± 0.8	11.5 ± 5.3
DE_expt1	Yellow	8.28 ± 0.09	1.25 ± 0.33	35.0 ± 1.3	13.7 ± 4.9
Oh40B	Yellow	6.14 ± 0.62	1.09 ± 0.04	22.3 ± 1.8	18.5 ± 0.6
CML52	Yellow	7.36 ± 2.27	1.06 ± 0.69	32.2 ± 9.9	13.8 ± 9.3
Tx303	Yellow	5.31 ± 1.05	0.83 ± 0.05	43.0 ± 8.6	17.1 ± 1.2
CML228	Yellow	8.14 ± 1.65	1.56 ± 0.50	26.5 ± 5.3	14.7 ± 4.5
CHZM 05 003	Yellow	11.79 ± 2.55	1.20 ± 0.40	44.7 ± 9.7	15.8 ± 5.6
Little Red Flint	Yellow	8.49 ± 0.44	1.57 ± 0.53	30.5 ± 1.3	17.2 ± 6.5
John Haulk	Yellow	7.54 ± 1.91	1.09 ± 0.76	29.6 ± 7.3	13.3 ± 9.3
Jackie Freeman	Yellow	6.61 ± 0.46	1.01 ± 0.11	27.1 ± 2.4	14.5 ± 1.0
Brittain Flint	Yellow	9.90 ± 0.06	1.72 ± 0.53	31.3 ± 1.9	13.6 ± 2.3
PHW17	White	6.40 ± 1.41	0.61 ± 0.15	28.0 ± 6.1	9.09 ± 1.9
PHT60	White	7.36 ± 1.21	0.97 ± 0.40	24.9 ± 3.3	14.5 ± 6.2
CML103	White	6.67 ± 1.50	1.13 ± 0.53	38.0 ± 9.1	16.4 ± 7.7
CML247	White	7.72 ± 1.76	1.89 ± 0.55	48.5 ± 14.9	26.0 ± 8.0
CML333	White	7.61 ± 0.61	1.20 ± 0.25	16.4 ± 1.9	8.47 ± 2.9
M37W	White	8.04 ± 0.58	1.53 ± 0.10	37.0 ± 4.1	18.9 ± 2.4
CML158Q	White	8.74 ± 1.06	1.79 ± 0.27	55.0 ± 6.5	35.1 ± 8.2
Olotillo Blanco	White	7.44 ± 1.11	1.27 ± 0.33	36.5 ± 5.5	16.8 ± 4.4
Ahumado	White	7.15 ± 1.01	0.93 ± 0.48	34.5 ± 5.6	14.3 ± 7.2
Z18-007	White	8.21 ± 0.97	1.86 ± 0.13	32.9 ± 3.3	24.0 ± 3.5
Andaqui	White	8.86 ± 0.01	1.68 ± 0.22	28.7 ± 1.4	14.9 ± 2.1
Cherokee Flour	White	6.69 ± 1.31	0.54 ± 0.05	27.6 ± 4.8	10.1 ± 1.9
Harinoso de Ocho	White	8.34 ± 2.28	0.99 ± 0.49	30.7 ± 8.1	10.6 ± 6.4
White Gourdseed	White	9.92 ± 1.31	1.28 ± 0.35	46.9 ± 6.1	22.0 ± 6.1
Burris	White	7.28 ± 0.17	1.16 ± 0.14	28.5 ± 1.1	16.9 ± 1.6
Southern Beauty	White	6.71 ± 1.51	1.16 ± 0.50	31.4 ± 9.1	13.4 ± 5.8
NC Shoepeg	White	8.37 ± 0.63	1.60 ± 0.04	38.3 ± 2.7	27.0 ± 1.1
Jimmy Red	Red	8.50 ± 0.84	1.46 ± 0.36	28.0 ± 2.8	13.4 ± 3.8
Chapalote	Brown	9.14 ± 1.85	1.81 ± 1.12	31.4 ± 6.2	16.7 ± 11.0
Ohio Blue Clarage	Blue	6.15 ± 1.99	0.77 ± 0.65	29.2 ± 10.0	8.82 ± 9.8
Maiz Morado	Black	6.94 ± 2.69	0.46 ± 0.22	24.6 ± 10.3	6.05 ± 2.9
Apache	Black	11.37 ± 1.64	1.33 ± 0.44	31.9 ± 5.0	12.5 ± 3.3
Cabuya Amarillo	Mix	8.12 ± 1.57	1.66 ± 0.27	29.2 ± 4.8	11.9 ± 1.8
Cariaco-Costeno	Mix	6.28 ± 0.06	1.21 ± 0.91	23.6 ± 0.2	16.8 ± 14.3
Kulli	Mix	7.88 ± 0.36	1.19 ± 0.25	28.8 ± 2.2	12.1 ± 2.1
PA Butter Flavor	Mix	9.08 ± 0.65	1.65 ± 0.16	32.1 ± 3.0	17.4 ± 2.3
Ancho King	Mix	7.69 ± 0.48	1.12 ± 0.66	24.1 ± 1.8	10.2 ± 6.4
Lail Flint	Mix	7.29 ± 1.47	1.15 ± 0.20	24.8 ± 5.5	15.4 ± 2.9

[1]Values are means ± SD of two measurements from each genotype after cooking. Phytate concentrations are expressed as milligrams per gram of cooked, lyophilized and milled maize sample (dry weight).

Supplementary Table S3. Zinc concentrations and phytate to zinc molar ratios of the Maize Nutrition Panel before and after degermination.[1]

		Zinc (µg/g)		Phytate – Zinc Molar Ratio	
Genotype	Color	Whole	Degerminated	Whole	Degerminated
Pioneer 3245	Yellow	13.88 ± 1.05	3.93 ± 0.24	36.4 ± 5.9	26.4 ± 5.4
LH54	Yellow	32.10 ± 0.24	10.45 ± 0.07	20.8 ± 2.2	16.3 ± 0.8
LH57	Yellow	31.73 ± 0.43	11.18 ± 0.13	23.6 ± 4.7	8.5 ± 0.5
LH196	Yellow	19.70 ± 0.01	5.49 ± 0.31	32.1 ± 6.6	27.0 ± 4.9
LH205	Yellow	16.68 ± 0.65	5.05 ± 0.12	36.0 ± 5.0	19.1 ± 14.0
PHG83	Yellow	20.82 ± 0.09	8.53 ± 0.49	33.5 ± 1.6	17.2 ± 2.8
PHM49	Yellow	25.44 ± 0.66	7.69 ± 0.30	30.1 ± 0.9	18.0 ± 3.0
MBST	Yellow	32.92 ± 0.43	11.98 ± 0.10	17.9 ± 9.0	4.6 ± 1.1
S8326	Yellow	26.39 ± 0.17	7.26 ± 0.13	26.5 ± 11.4	14.3 ± 11.6
W64A	Yellow	23.43 ± 0.04	6.09 ± 0.26	29.7 ± 0.8	10.1 ± 2.1
Mo47	Yellow	18.83 ± 0.20	6.06 ± 0.16	42.1 ± 0.4	16.2 ± 10.8
H100	Yellow	19.71 ± 0.39	6.47 ± 0.11	37.1 ± 8.6	14.6 ± 3.2
A619	Yellow	30.80 ± 0.93	11.20 ± 0.04	22.1 ± 4.0	12.2 ± 0.9
Pa875	Yellow	27.43 ± 0.04	10.91 ± 0.07	23.5 ± 1.0	10.6 ± 4.2
DE_expt1	Yellow	26.33 ± 0.27	9.59 ± 0.23	31.0 ± 0.0	12.9 ± 3.1
Oh40B	Yellow	24.25 ± 0.15	8.79 ± 0.11	24.9 ± 2.4	12.2 ± 0.6
CML52	Yellow	17.41 ± 0.29	5.82 ± 0.15	41.6 ± 12.1	18.0 ± 12.1
Tx303	Yellow	16.67 ± 0.17	6.84 ± 0.23	31.4 ± 6.6	11.9 ± 0.3
CML228	Yellow	20.76 ± 0.24	8.71 ± 0.12	38.6 ± 8.3	17.6 ± 5.4
CHZM 05 003	Yellow	22.85 ± 0.06	7.35 ± 0.08	50.8 ± 10.9	16.1 ± 5.2
Little Red Flint	Yellow	27.69 ± 0.46	7.10 ± 0.21	30.2 ± 1.1	21.8 ± 6.7
John Haulk	Yellow	27.25 ± 0.08	9.90 ± 0.16	27.3 ± 7.0	10.9 ± 7.8
Jackie Freeman	Yellow	23.67 ± 0.63	6.80 ± 0.08	27.5 ± 2.6	14.6 ± 1.8
Brittain Flint	Yellow	34.29 ± 0.33	12.26 ± 0.05	28.4 ± 0.4	13.8 ± 4.3
PHW17	White	24.40 ± 0.55	5.50 ± 0.01	25.9 ± 5.1	11.0 ± 2.6
PHT60	White	29.17 ± 1.05	6.28 ± 0.04	24.9 ± 3.2	15.3 ± 6.1
CML103	White	14.17 ± 0.15	6.46 ± 0.44	46.4 ± 10.9	17.2 ± 9.2
CML247	White	20.95 ± 0.02	8.35 ± 0.24	36.3 ± 8.3	22.3 ± 7.2
CML333	White	21.54 ± 0.66	5.57 ± 0.27	34.8 ± 1.7	21.3 ± 3.3
M37W	White	19.67 ± 0.49	7.72 ± 0.05	40.2 ± 3.9	19.5 ± 1.5
CML158Q	White	18.68 ± 0.12	7.26 ± 0.04	46.1 ± 5.9	24.3 ± 3.5
Olotillo Blanco	White	23.34 ± 0.43	8.53 ± 0.06	31.4 ± 5.3	14.6 ± 3.9
Ahumado	White	21.22 ± 0.75	5.64 ± 0.10	33.2 ± 5.9	16.3 ± 8.2
Z18-007	White	27.80 ± 0.83	9.22 ± 0.05	29.1 ± 2.6	19.9 ± 1.5
Andaqui	White	23.37 ± 0.24	7.40 ± 0.04	37.4 ± 0.4	22.4 ± 2.8
Cherokee Flour	White	28.29 ± 0.70	7.99 ± 0.07	23.3 ± 4.0	6.7 ± 0.6
Harinoso de Ocho	White	20.10 ± 0.08	6.24 ± 0.11	40.9 ± 11.0	16.0 ± 7.5
White Gourdseed	White	26.83 ± 0.13	7.83 ± 0.12	36.4 ± 5.0	16.1 ± 4.7
Burris	White	23.71 ± 0.11	6.91 ± 0.20	30.2 ± 0.8	16.5 ± 2.5
Southern Beauty	White	20.57 ± 1.34	7.78 ± 0.01	32.1 ± 9.3	14.7 ± 6.3
NC Shoepeg	White	25.40 ± 0.70	6.94 ± 0.06	32.5 ± 3.3	22.7 ± 0.5
Jimmy Red	Red	26.88 ± 0.10	7.32 ± 0.04	31.1 ± 3.2	19.7 ± 4.7
Chapalote	Brown	24.61 ± 0.02	6.81 ± 0.41	36.6 ± 7.4	26.2 ± 17.8
Ohio Blue Clarage	Blue	21.16 ± 0.25	7.36 ± 1.21	28.6 ± 9.6	10.2 ± 10.5
Maiz Morado	Black	26.05 ± 0.35	9.46 ± 0.01	26.2 ± 10.5	4.8 ± 2.3
Apache	Black	36.66 ± 0.16	9.27 ± 0.29	30.5 ± 4.3	14.2 ± 4.2
Cabuya Amarillo	Mix	25.20 ± 0.20	11.11 ± 0.18	31.7 ± 5.9	14.7 ± 2.2
Cariaco-Costeno	Mix	22.27 ± 0.02	6.78 ± 0.05	27.8 ± 0.3	17.5 ± 13.1
Kulli	Mix	23.59 ± 0.15	6.63 ± 0.18	32.9 ± 1.7	17.7 ± 4.2
PA Butter Flavor	Mix	29.42 ± 0.09	9.74 ± 0.19	30.4 ± 2.3	16.7 ± 2.0
Ancho King	Mix	31.06 ± 0.43	10.22 ± 0.07	24.4 ± 1.9	10.8 ± 6.4
Lail Flint	Mix	25.66 ± 0.33	8.32 ± 0.04	28.0 ± 6.0	13.6 ± 2.4

[1]Values are means ± SD of two measurements from each genotype after cooking. Zinc concentrations are expressed as micrograms per gram of cooked, lyophilized and milled maize sample (dry weight).

Supplementary Table S4. Calcium and magnesium concentrations of the Maize Nutrition Panel before and after degermination.[1]

Genotype	Color	Calcium (µg/g) Whole	Calcium (µg/g) Degerminated	Magnesium (µg/g) Whole	Magnesium (µg/g) Degerminated
Pioneer 3245	Yellow	29.81 ± 5.36	16.70 ± 2.22	821.3 ± 2.5	146.2 ± 12.6
LH54	Yellow	67.93 ± 1.99	25.33 ± 1.06	944.5 ± 1.2	217.9 ± 0.5
LH57	Yellow	62.93 ± 0.51	39.47 ± 2.57	1001.1 ± 2.2	270.8 ± 1.0
LH196	Yellow	35.06 ± 3.93	19.67 ± 2.19	895.4 ± 7.7	122.1 ± 0.3
LH205	Yellow	22.79 ± 0.03	14.83 ± 0.28	697.6 ± 52.3	100.7 ± 1.7
PHG83	Yellow	31.71 ± 3.08	18.38 ± 1.79	907.2 ± 23.7	197.5 ± 4.4
PHM49	Yellow	29.30 ± 1.06	16.46 ± 2.15	999.6 ± 20.3	181.9 ± 0.2
MBST	Yellow	42.68 ± 3.00	32.26 ± 1.39	982.9 ± 1.9	202.5 ± 1.7
S8326	Yellow	50.70 ± 2.55	21.99 ± 0.46	997.2 ± 3.9	194.6 ± 0.1
W64A	Yellow	28.55 ± 0.36	13.50 ± 2.45	1051.2 ± 7.1	153.0 ± 0.2
Mo47	Yellow	38.99 ± 0.49	20.60 ± 0.37	1030.9 ± 7.7	158.6 ± 0.9
H100	Yellow	24.43 ± 2.26	15.08 ± 0.14	1022.1 ± 0.1	185.6 ± 3.9
A619	Yellow	19.72 ± 0.74	25.07 ± 0.97	964.7 ± 37.4	259.1 ± 1.5
Pa875	Yellow	37.94 ± 1.49	18.25 ± 0.71	933.2 ± 0.4	198.6 ± 0.7
DE_expt1	Yellow	53.74 ± 0.85	29.93 ± 0.13	1036.1 ± 7.1	206.6 ± 0.2
Oh40B	Yellow	32.10 ± 1.81	20.82 ± 0.14	818.0 ± 12.9	178.6 ± 0.3
CML52	Yellow	33.11 ± 1.44	16.02 ± 0.29	874.7 ± 0.6	163.4 ± 0.9
Tx303	Yellow	29.39 ± 3.24	15.11 ± 0.69	830.5 ± 6.9	248.6 ± 0.7
CML228	Yellow	34.11 ± 0.75	16.73 ± 0.68	1077.9 ± 8.3	251.9 ± 1.5
CHZM 05 003	Yellow	40.02 ± 1.77	28.57 ± 0.08	1165.3 ± 18.3	199.4 ± 3.8
Little Red Flint	Yellow	76.56 ± 1.10	39.48 ± 5.31	1134.2 ± 16.5	180.8 ± 0.4
John Haulk	Yellow	34.98 ± 0.30	26.91 ± 2.79	1029.5 ± 18.5	211.6 ± 2.6
Jackie Freeman	Yellow	43.62 ± 1.48	23.13 ± 0.67	997.5 ± 21.6	137.7 ± 0.6
Brittain Flint	Yellow	43.74 ± 1.57	23.10 ± 0.74	1229.5 ± 5.2	245.3 ± 4.3
PHW17	White	21.64 ± 1.44	11.90 ± 0.14	916.2 ± 3.6	155.0 ± 0.7
PHT60	White	47.25 ± 5.50	28.07 ± 6.24	983.6 ± 3.9	129.3 ± 2.7
CML103	White	53.20 ± 0.36	33.01 ± 1.82	956.5 ± 2.8	226.0 ± 0.8
CML247	White	57.15 ± 2.23	31.13 ± 0.97	934.4 ± 14.5	263.5 ± 7.9
CML333	White	63.96 ± 2.69	27.43 ± 0.28	997.9 ± 3.9	199.9 ± 0.9
M37W	White	108.47 ± 23.92	31.17 ± 1.50	1004.7 ± 24.6	198.0 ± 2.4
CML158Q	White	60.54 ± 0.01	26.59 ± 0.17	956.8 ± 8.3	194.5 ± 1.9
Olotillo Blanco	White	34.59 ± 1.48	18.57 ± 0.34	945.1 ± 8.4	200.3 ± 1.5
Ahumado	White	48.79 ± 8.30	21.45 ± 0.49	871.2 ± 22.7	152.6 ± 1.2
Z18-007	White	136.58 ± 52.37	24.80 ± 0.54	924.8 ± 1.2	234.7 ± 0.4
Andaqui	White	66.73 ± 7.11	27.05 ± 0.79	1089.7 ± 12.0	253.1 ± 2.2
Cherokee Flour	White	30.02 ± 1.50	18.51 ± 0.19	934.6 ± 14.1	148.9 ± 1.7
Harinoso de Ocho	White	62.10 ± 0.18	37.75 ± 0.58	1038.6 ± 0.8	205.5 ± 0.8
White Gourdseed	White	52.12 ± 0.89	24.79 ± 0.58	1017.6 ± 0.5	188.4 ± 2.1
Burris	White	40.98 ± 1.30	28.52 ± 2.15	960.7 ± 6.5	163.6 ± 4.7
Southern Beauty	White	38.65 ± 0.76	22.59 ± 0.92	961.3 ± 69.1	213.5 ± 0.4
NC Shoepeg	White	66.35 ± 0.92	36.50 ± 1.84	1058.3 ± 6.7	197.0 ± 0.2
Jimmy Red	Red	44.98 ± 0.17	33.63 ± 1.16	1055.2 ± 11.5	160.5 ± 0.7
Chapalote	Brown	74.76 ± 2.26	49.76 ± 4.56	1326.8 ± 9.1	288.7 ± 7.0
Ohio Blue Clarage	Blue	47.52 ± 0.17	27.46 ± 0.05	908.3 ± 18.2	157.6 ± 1.3
Maiz Morado	Black	41.49 ± 0.15	27.91 ± 0.81	932.2 ± 41.5	150.8 ± 1.9
Apache	Black	47.42 ± 17.14	29.63 ± 11.53	1250.3 ± 0.4	149.8 ± 1.9
Cabuya Amarillo	Mix	49.25 ± 0.18	26.19 ± 0.54	1042.6 ± 3.2	211.3 ± 1.1
Cariaco-Costeno	Mix	49.97 ± 9.38	20.68 ± 1.12	1060.4 ± 3.7	145.2 ± 4.1
Kulli	Mix	67.29 ± 3.80	43.09 ± 1.94	999.1 ± 4.1	171.3 ± 2.0
PA Butter Flavor	Mix	51.38 ± 0.50	35.49 ± 0.13	1246.0 ± 0.6	222.7 ± 0.6
Ancho King	Mix	62.62 ± 0.46	37.53 ± 0.24	1107.5 ± 19.2	169.9 ± 1.7
Lail Flint	Mix	43.26 ± 1.44	27.94 ± 0.90	1092.4 ± 5.8	139.3 ± 0.5

[1]Values are means ± SD of two measurements from each genotype after cooking. Calcium and magnesium concentrations are expressed as micrograms per gram of cooked, lyophilized and milled maize sample (dry weight).

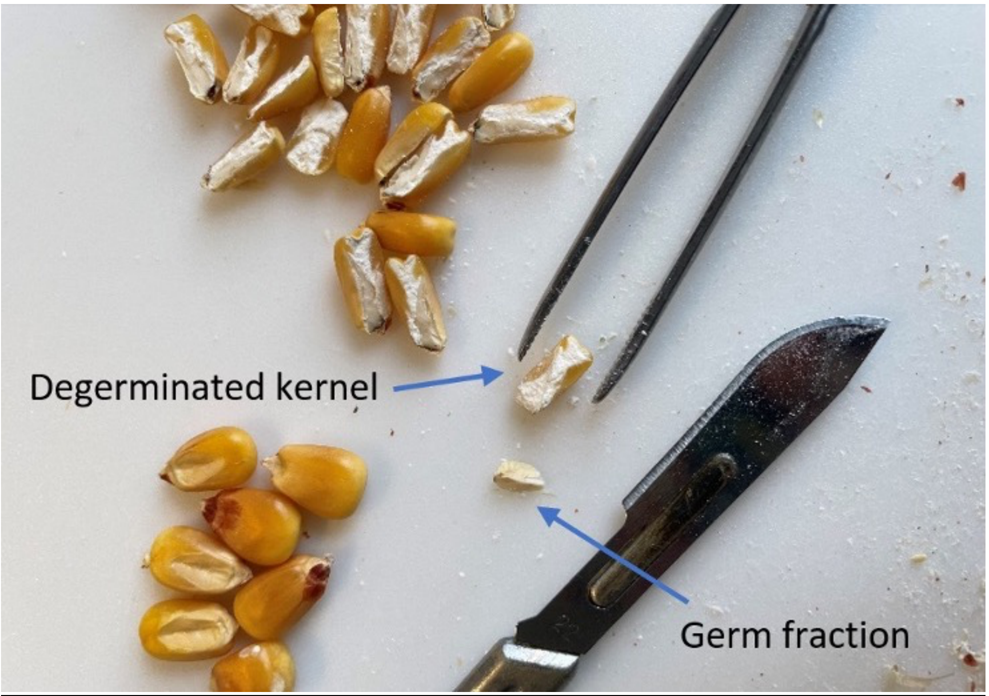

Supplementary Figure S2. Degermination of maize (Pioneer 3425) by hand as described by Glahn et al. (2019).

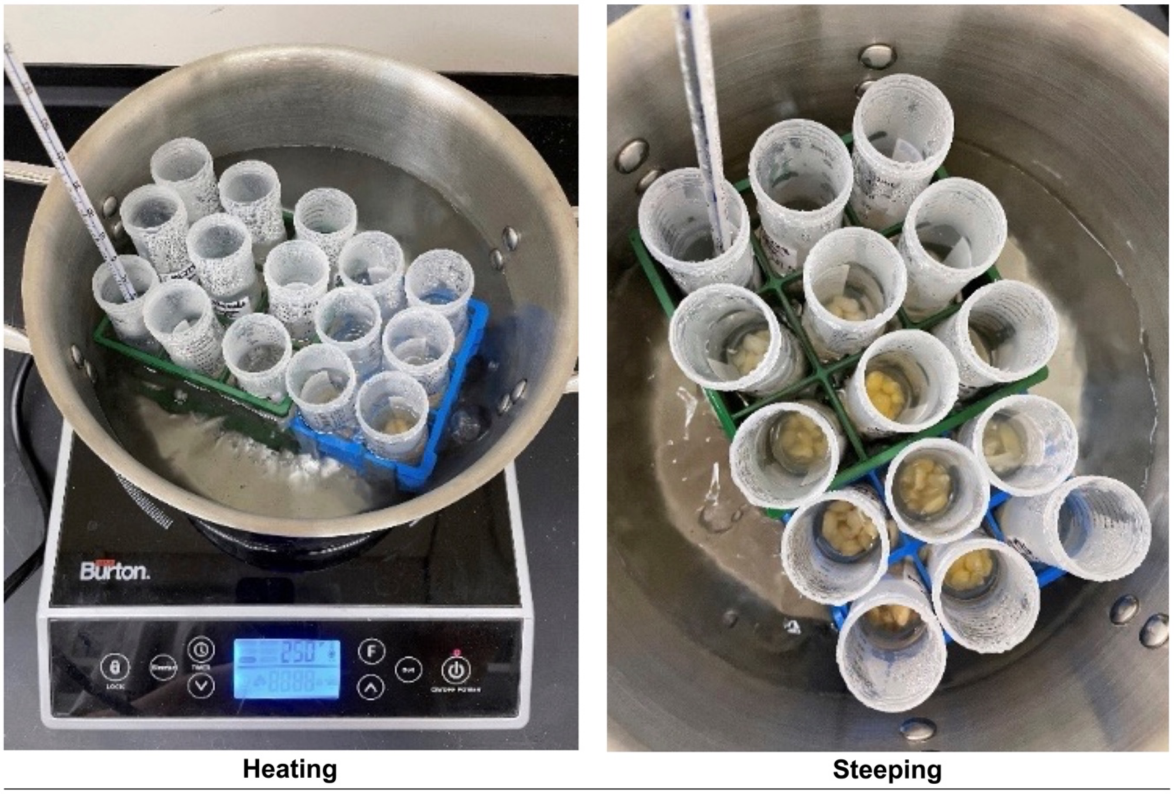

Supplementary Figure S3. Whole and degerminated maize samples were heated from 20ºC to 92ºC at a rate of 2.3ºC/min, cooked at 92ºC for 60 minutes, then heat was turned off and samples steeped for 3 hours until they returned to 25ºC.
